# Reliable evaluation method of heating power of magnetic nanofluids to directly predict the tumor temperature during hyperthermia

**DOI:** 10.1038/s41598-021-01321-3

**Published:** 2021-11-11

**Authors:** Ji-wook Kim, Seongtae Bae

**Affiliations:** 1 Nanobiomagentics and Bioelectronics Laboratory (NB2L), Department of Electrical Engineering, University of South Carolina, 301 Main Street, Columbia, SC 29208 USA; 2 Biomedical Engineering Program, College of Engineering and Computing, University of South Carolina, 301 Main Street, Columbia, SC 29208 USA

**Keywords:** Biomedical engineering, Nanobiotechnology, Nanoscale materials, Techniques and instrumentation

## Abstract

Reliable measurement of heating power of magnetic nanofluids (MNs) to accurately predict the AC heat-induction performance in tumors is highly desirable for clinical magnetic nanofluids hyperthermia (MNFH) application because it can save time for screening the performance of newly developed MNFH agent and minimize the over-use of animals dramatically. Here, a bio-mimicking phantom model, called Pseudo-Tumor Environment System (P-TES), biochemically designed by considering the external and internal critical factors related to the complex biological environments is proposed to provide a highly reliable evaluation method of heating performance of MNs for in-vivo MNFH applications. According to the experimentally analyzed results, the heating power of MNs measured using the P-TES is well accorded with the heating temperature measured in the tumors during in-vivo MNFH. This result strongly demonstrates that the proposed P-TES can be recommended as a standardized measurement method of heating performance of MNs for clinical MNFH application.

## Introduction

Magnetic nanofluid hyperthermia (MNFH) has been recently considered as an emerging modality for cancer treatment due to its non-invasiveness, minimized side effects, and high treatment efficacies^1–3^. It uses thermal energy induced by magnetic nanoparticles (MNPs) in response to an AC magnetic field, ${\text{H}}_{\text{AC,appl}}$, to destroy cancer cells^4,5^. For the last two decades, a huge effort has been devoted to the development of new MNPs with superior heat induction power by tuning the material’s parameters such as size, shape, and composition. Many studies have reported their successful applications to MNFH agents in biomedical fields^2,6–8^.

**Figure 1:**
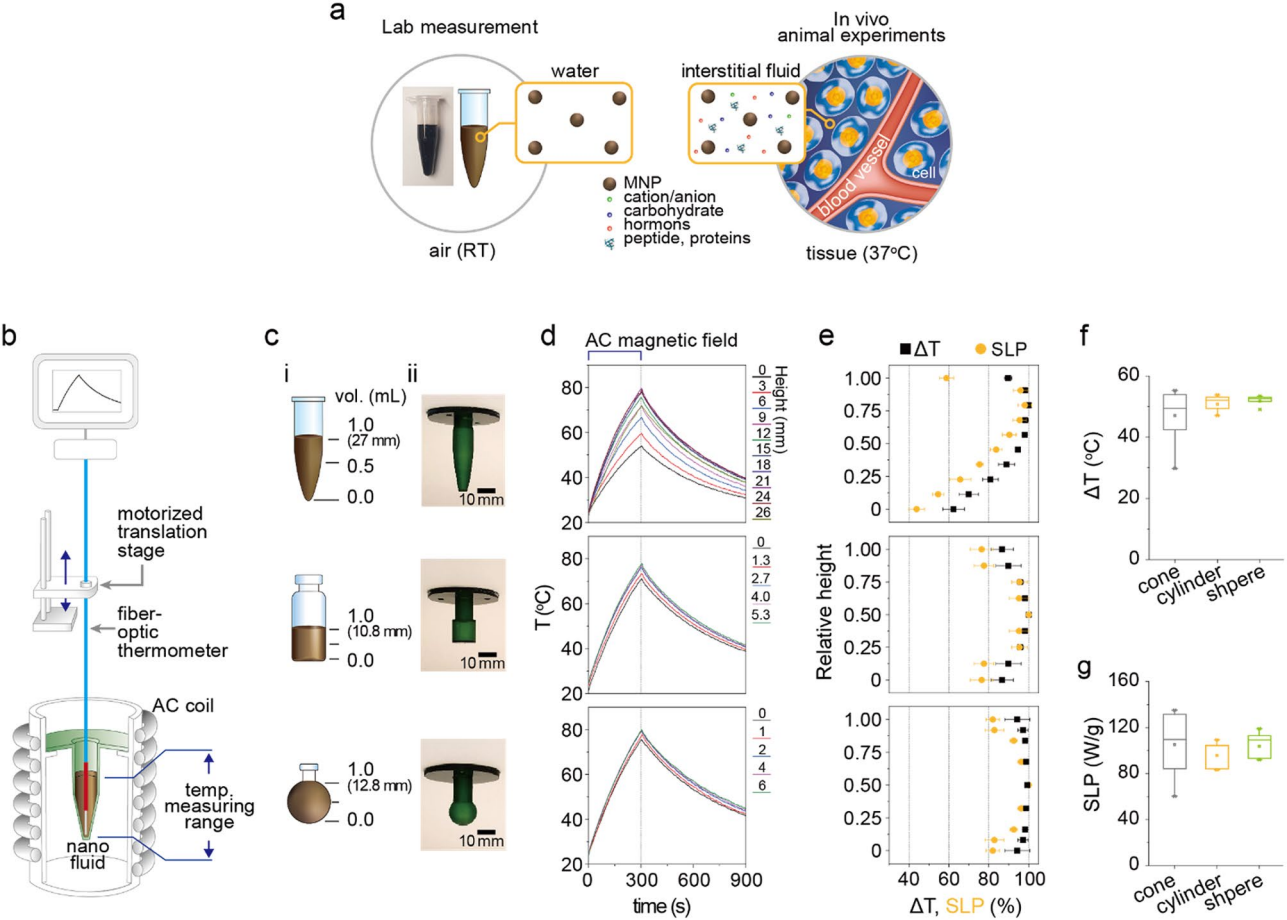
Comparison of heat induction measurement conditions and effects of sample container shape on the ΔT and SLP of magnetic nanofluids (MNs) during AC heat induction. (**a**) A schematic illustration showing the experimental conditions of heat induction measurement of MNs at the optimal laboratory (left) and in vivo (right) environments. MNs are dispersed in the interstitial fluid that contains various biological components including cation/anion, carbohydrate, hormones, and peptides. Due to these conditions, the viscosity, pH, and salt concentration of the dispersion medium, which are critical parameters to change the heat induction power of MNs, can be different from deionized water. In addition, the temperature of tissues is tightly regulated by temperature homeostasis (thermoregulation) in the body. (**b**) A schematic illustration of the experimental setup for measuring the temperature distribution in a sample container. (**c**) Illustrations (i) and pictures (ii) of cone- (top), cylindrical- (middle), and spherical- (bottom) shape sample containers. The volume of MNs were fixed at 1 mL. (**d**,**e**) The AC heating temperature rising characteristics (**d**), ΔT (black), and SLP (orange) distribution of Mg doped γFe_2_O_3_ MN measured at different heights (**e**). ΔT and SLP of MNs were rescaled to 100%. (**f**,**g**) The distribution of ΔT (**f**) and SLP (**g**) obtained from each sample container.

The most general term that expresses the heating ability of magnetic nanofluids (MNs) is specific loss power (SLP). It is defined as the heating power generated per unit mass (W g^−1^) and experimentally determined from the heating-up rate of MNs (dT/dt; T, temperature; t, time) at a given amount of MNPs (m), $\text{SLP }= \text{ } \frac{\text{CV}}{{\text{m}}}\frac{\text{dT}}{{\text{dt}}}$ (*V*: sample volume, C: heat capacity of solvent)^4,9^. In the most experimental conditions (i.e., non-adiabatic condition), an initial slop of the magnetic heating curve (e.g., the first 30 s of *dT/dt*) is used for SLP measurement to get rid of the external heat transfer effect^4^. Although the SLP generally represents the intrinsic heat performance of MN, it is still difficult to accurately predict the heating ability at the biological conditions, specifically, tissue temperature, based on the SLP measured at the optimal laboratory conditions. Therefore, the determination of therapeutically sufficient concentration of MNs is still difficult, thus several in-vivo performance tests are additionally required to evaluate the heating capability in biological medium. This is because the currently used SLP measurement method does not consider the complex biological conditions such as total ionic concentration, pH, and viscosity, etc. (Fig. 1a)^10,11^ directly or indirectly affecting the relaxation time constant of MNPs. The interaction change of MNPs with biological medium or its-induced MNPs aggregations^12–15^ causes the change of relaxation time constant, which is a critical physical parameter in determining the heating power at the ${\text{H}}_{\text{AC,appl}}$. Additionally, the body temperature and blood perfusion make it more difficult to estimate the tissue (tumor tissue) temperature during MNFH due to the active cooling mechanism (i.e., homeostasis), which is not considered in the current SLP measurement.

In this study, we investigated the external and internal factors that can critically affect the heat induction performance of MNs in biological conditions and established a bio-mimicking phantom model to provide an accurate and reliable heat induction power evaluation method for clinical MNFH application. The effects of external experimental and environmental factors on the heating power of MNs were first explored. Particularly, various sample containers with different shapes were tested to remove the measurement uncertainty associated with the shape of sample containers and to find an optimal container because the container can be a basic experimental error-making factor in measuring the heating power of MNs. Secondly, the effects of surrounding medium (SM) on the heating power of MNs were systematically investigated in the air, 37 °C water, and 37 °C water with different flow rates (0 ~ 0.5 mL mg^−1^ min^−1^) to reflect the heat transfer effect of human body and accurately estimate the AC heating temperature rise (ΔT) in the biological environments. Thirdly, the effects of internal experimental parameters such as salt concentration, pH, and viscosity on the heating power of MNs were investigated. By introducing glycerol/agarose, pH 5 ~ 7, and 150 mM NaCl to a phantom model, the colloidal stability and the Brownian relaxation time-dependent change of heating power of MNs were simultaneously evaluated. Finally, the technical validity of the proposed bio-mimicking phantom model was verified using the tumor xenografted mouse models by in-vivo MNFH studies.

## Results and discussion

### Sample container effect

**Figure 2:**
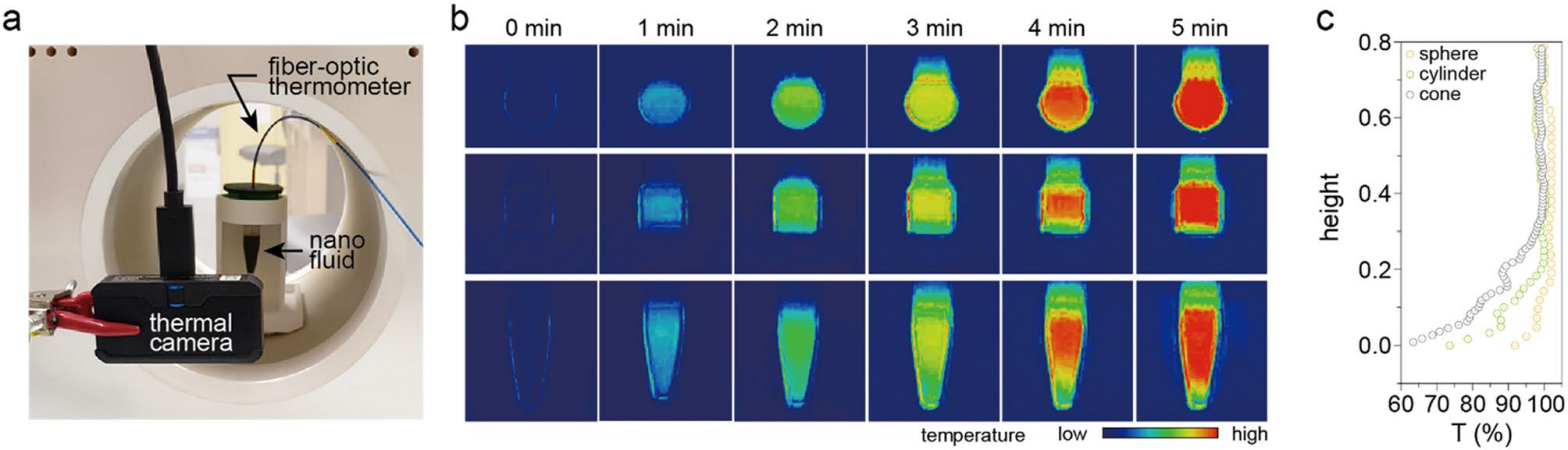
The surface temperature measurement of different sample containers during AC heat induction. (**a**) A picture of the experimental setup. (**b**) The thermal images of spherical- (top), cylindrical- (middle), and cone (bottom)- shape sample containers during application of AC magnetic field (*f*_*appl*_ = 100 kHz, *H*_*appl*_ = 140 Oe) for 5 min. (**c**) The surface temperature distribution obtained from thermal images of each sample container. The 100% of temperature represents the maximum temperature measured at each sample container.

For the precise and reproducible heat measurement of MNs, the relationship between the temperature distribution of MNs across the sample container and the shape of the sample container was systematically investigated. To find an optimal sample container for SLP measurement, three representative sample containers with cone, cylindrical, and spherical-shapes were prepared and the local temperature of MNs at the different heights in the containers (Fig. 1b) was measured. All sample containers were fabricated using a UV resin 3D printer and the internal volume was fixed at 1 mL (Fig. 1c). The height-dependent temperature of MNs was monitored using a fiber-optic thermometer mounted to a motorized vertical lift stage (Fig. 1b and Supplementary Fig. 1). This system allows to precisely control the height in the vertical direction. The 25 nm Mg doped γFe_2_O_3_ MNs showing superior AC induction heating performance^2,8^ were used as a testing MN (Supplementary Figs. 2 and 3) and the ${\text{H}}_{\text{AC,appl}}$ (*f*_appl_·*H*_appl_ < 5.0 × 10^9^ Am^−1^ s^−1^ or *f*_appl_ < 120 kHz, *H*_appl_ < 190 Oe)^16,17^ was applied to the MNs (10 mg mL^−1^) to measure the AC heating characteristics. The wide variation of AC heat induction curves depending on the position of temperature probe was observed for the cone-shape container compared to other containers (Fig. 1d). Specifically, the cone-shape container showed the largest temperature difference between the top (78 °C) and the bottom (51 °C) positions. Additionally, it had the same variation characteristics in ΔT and calculated SLP (Fig. 1e) depending on the probe position. A large deviation of ΔT (19%, ± 8.7 °C) and SLP (27%, ± 25.4 W g^−1^) was observed for the cone-shape container (Fig. 1f,g). While, the smallest deviation of ΔT (3%, ± 1.7 °C) and SLP (11%, ± 10.6 W g^−1^) was observed for the spherical-shape container. The temperature distribution was further investigated using thermal imaging of each container during MNFH. When the surface temperature of sample containers was monitored using a thermal camera at the ${\text{H}}_{\text{AC,appl}}$ (Fig. 2a, Supplementary Fig. 4), the inhomogeneous temperature distribution along the vertical direction was observed for cylindrical- and cone-shape containers, but the sphere-shape container showed temperature distribution with a higher homogeneity (Fig. 2b,c). The different local heat loss rates depending on the position of cylindrical- and cone-shape containers due to the irregular surface area would be critically responsible for the large inhomogeneous temperature distribution^18^. These results indicate that the shape of sample container should be considered as one of the critical factors in accurately evaluating the heating performance of newly developed MNs in lab-based experiments to estimate its validity for in-vivo MNFH application.

### Surrounding medium

**Figure 3:**
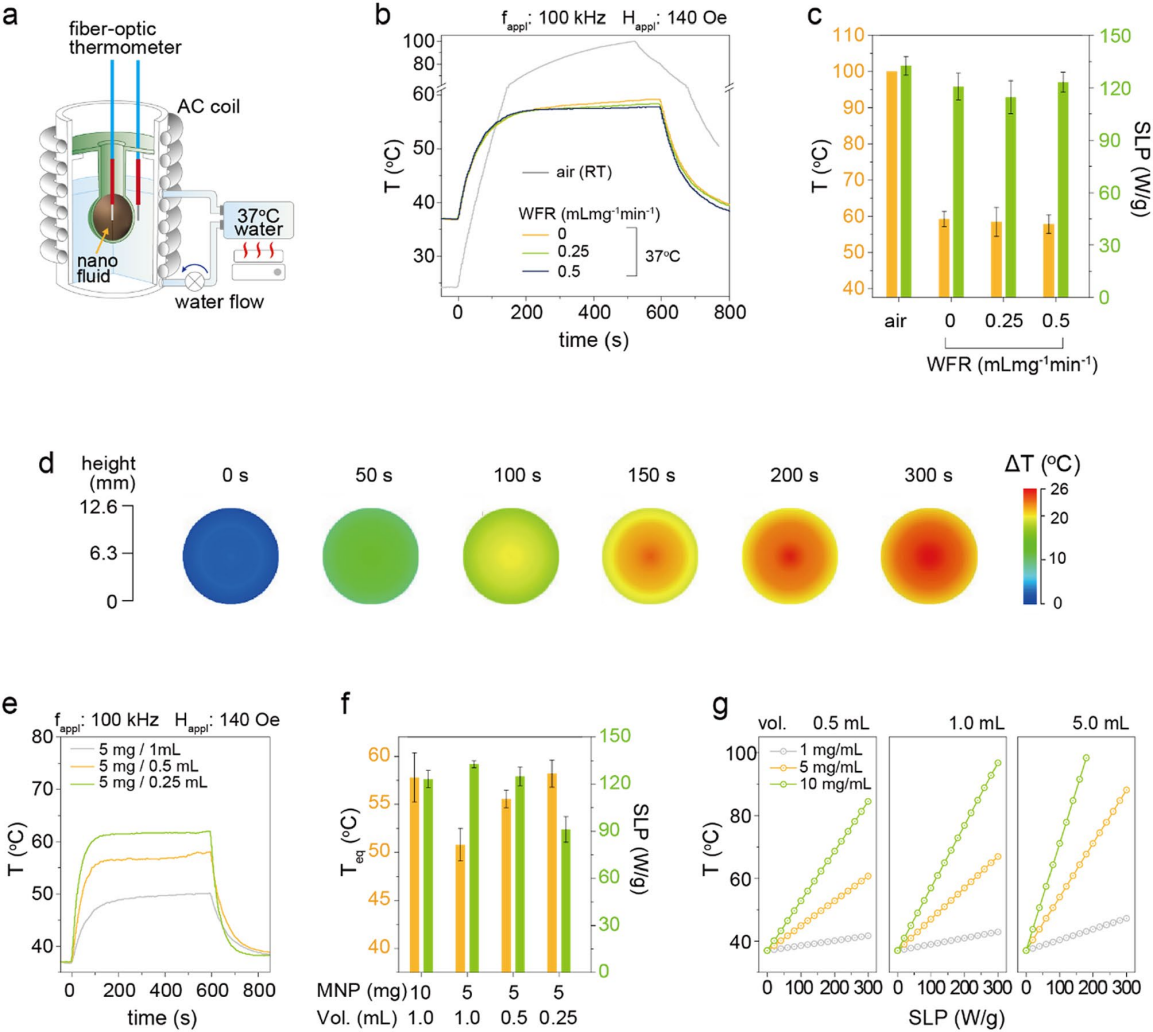
Effects of surrounding medium on the ΔT and SLP of MNs. (**a**) A schematic illustration of the experimental setup. The temperature and volume of surrounding water are 37 °C and 40 mL, respectively. The water flow rate (WFR) is controlled by a peristaltic pump. (**b**) The AC heating temperature rising characteristics of Mg doped γFe_2_O_3_ MNs measured at the different surrounding medium conditions of air (RT, 23 °C) and 37 °C water with 0, 0.25, and 0.5 mL mg^−1^ min^−1^ WFR. (**c**) The heating-up temperature (orange) and SLP (green) obtained from different surrounding medium conditions. (**d**) The temperature distribution of 1 mL Mg doped γFe_2_O_3_ MN in spherical-shape sample container during the application of AC magnetic field for 5 min. (WFR = 0.25 mL mg^−1^ min^−1^). (**e**–**g**) The heat transfer coefficient measurement and calculated equilibrium temperature. The AC heating temperature rising characteristics of Mg doped γFe_2_O_3_ MNs (**e**), equilibrium temperature (orange), and SLP (green) measured at the different concentrations (5–20 mg mL^−1^) and volumes (0.25–1.0 mL) (**f**). Calculated equilibrium temperature at the different concentrations and volumes as a function of SLP (**g**).

To investigate the effects of surrounding medium (SM), which is another external experimental factor, on the determination of ΔT and SLP of MNs, a circulating water bath system (40 mL) was established on the inside of AC magnetic coil (Fig. 3a, Supplementary Fig. 5a,b). This system is basically designed to mimic human body temperature and blood perfusion (0.1–1.0 mL mg^−1^ min^−1^) related to body temperature homeostasis^19^. Water was used as a SM instead of air which is typically used for SLP measurement because the body is mostly composed of water ($\approx$ 85%). The water bath was connected to a temperature controller to keep the temperature at 37 °C. The water flow rate (WFR) was controlled at 0, 0.25, and 0.5 mL mg^−1^ min^−1^ using a peristaltic pump. The sphere-shape sample container with Mg doped γFe_2_O_3_ MN (1 mL) was placed in the water bath (Fig. 3a). The temperature of MNs and SM were monitored using fiber-optic thermometers. The AC heat induction curve and maximum temperature (T_AC,max_) were measured from 10 mg mL^−1^ Mg doped γFe_2_O_3_ MNs and the corresponding SLP are shown in Fig. 3b,c. While the temperature of MNs in the air SM rapidly increased up to 100 °C (< 10 min), the three nanofluids placed in the water SM with different WFR were stably saturated around T_AC,max_ ≈ 57 °C. There was no obvious dependence of T_AC,max_ on the WFR and the calculated SLPs measured from Fig. 3b were lied in a similar range of 110–120 W g^−1^ regardless of the SM conditions and WFR. The heat transfer effects during the first 30 s would be very small because there is a negligible difference in the initial temperature between the MNs and the SM. Therefore, the initial slope (dT/dt = 0.29 K s^−1^ (air), 0.31 K s^−1^ (water), Fig. 3b) and the SLP for the three MNs were similar each other. However, the AC heating curve measured in the water SM reached at saturation temperature faster than the air condition due to the higher heat transfer coefficient of water [10^2^ ~ 10^3^ Wm^−2^ K^−1^, air (1 ~ 30 Wm^−2^ K^−1^)]^20^. The heat induction temperature distribution of MNs was further characterized in the water SM (Fig. 3d, Supplementary Fig. 6) to investigate the effects of heat sinking on the change of T_AC,max_ and SLP. The temperature was homogeneous throughout the whole sample container at the initial stage (0–50 s) owing to the negligibly small heat sinking. However, there was a 4 °C difference between the center and the peripheral region of MN at 300 s due to the heat sinking through the SM. These results indicate that the ΔT measured at the conventional lab conditions (i.e., air SM with RT) is not valid in characterizing the heating performance of MN in the biological environment due to the ambient condition-induced heating sinking effects. Therefore, the SLP and ΔT (T_AC,max_) of MNs measured in the water SM can be more representative for the biological (in-vivo) application. Regarding WFR effects, it caused a minimal temperature difference after saturation in our experiments (Fig. 3b), but it would expect to be influential in in-vivo environments due to organ and tissue dependent different blood perfusion rates.

The heat transfer from MN to water SM was further studied to establish a ΔT prediction model depending on SLP in the biological environments. The heat exchange to an environment is followed by the Newton-Richman law and the thermal energy balance is expressed by^21^,

1 $$\frac{{dE}}{{dt}}\left( {increase\;of\;thermal\;energy} \right) = W\left( {heat\;generation} \right) - W(heat\;sink),$$

where, *t* is time and *W* is power. The MN is considered as an internal heat source and the heat sinks to SM. When the temperature is reached at an equilibrium state, *T*_*eq*_, the heat generation and sinking are assumed to be equal, thus Eq. (1) can be rewritten by Eqs. (2) and (3).

2 $$W (nanofluid) = W (heat \,sink),$$

3 $$SLP \cdot m = \alpha F ( T_{eq} - T_{s} ),$$

where, *m* is mass of MNP, *α* is heat transfer coefficient, *F* is area of surface, and *T*_*s*_ is temperature of SM. Since the water SM is stably maintained at 37 °C during the application of ${\text{H}}_{\text{AC,appl}}$ (Supplementary Fig. 5c), *T*_*s*_ is assumed to be 37 °C. Therefore, the *T*_*eq*_ at the known SLP and mass can be determined if the heat transfer coefficient is found for the system. From the experimental results on *T*_*eq*_ and SLP measured at the various concentrations (1–20 mg mL^−1^) and volumes (0.25–1.0 mL) of MNs (Fig. 3e,f), the heat transfer coefficient for water SM was determined at 103.7 ± 7.3 W m^−2^‧K. Hence, the predicted *T*_*eq*_ (and ΔT) was reversely calculated as a function of SLP at the given volume and concentration (Fig. 3g).

### Physiological factors

**Figure 4:**
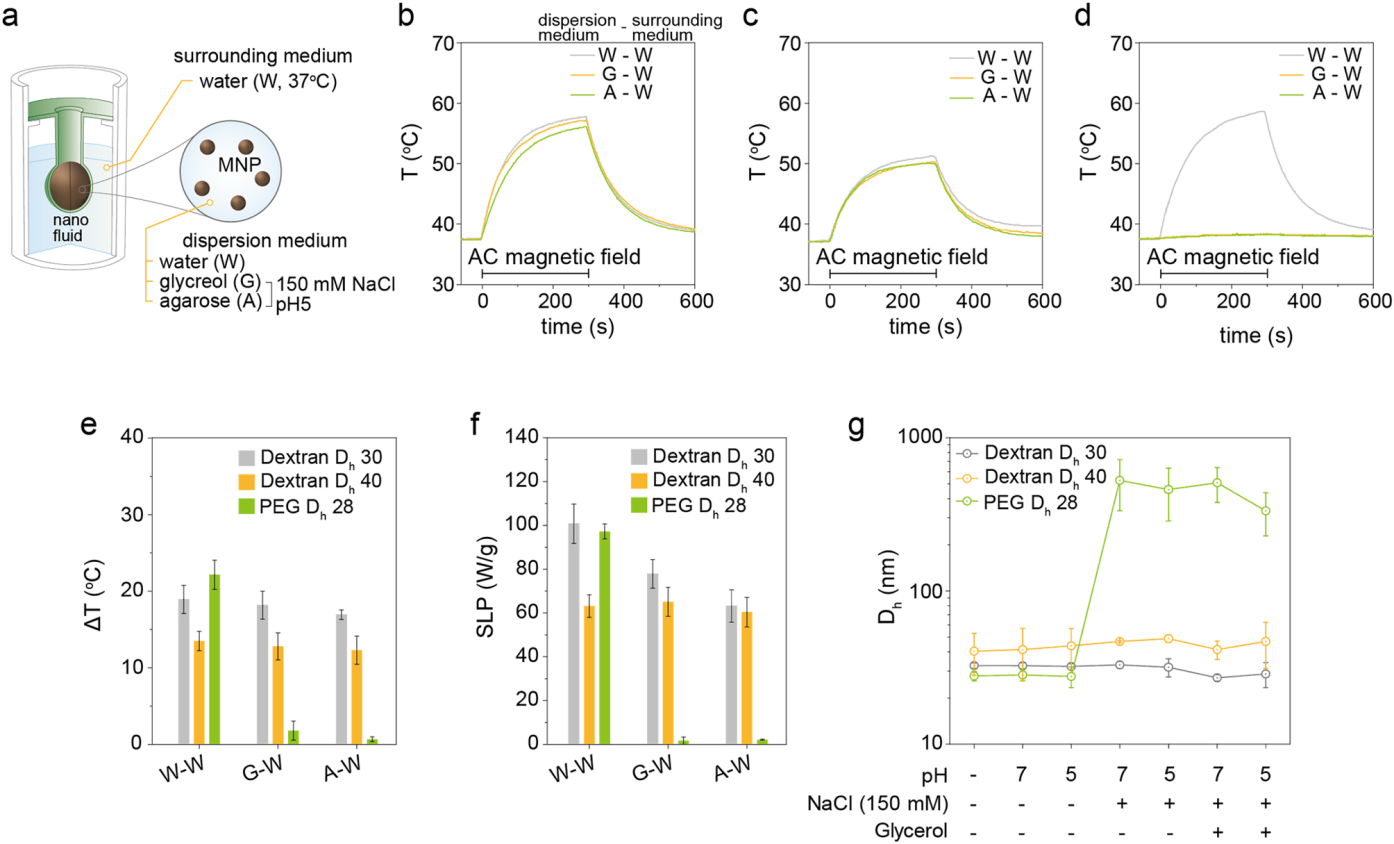
Effects of physiological conditions on the SLP and ΔT of MN during AC heat induction. (**a**) A schematic illustration of the experimental setup. Deionized water (W), glycerol (G), and agarose (A) with 150 mM NaCl and pH 5 were used as a dispersion medium for Mg doped γFe_2_O_3_ MNs. The temperature of surrounding air and water media were RT (23 °C) and 37 °C, respectively. (**b**–**d**) The AC magnetic heat induction curves of dextran-110 k (Dextran D_h_ 30, **b**), dextran-10 k (Dextran D_h_ 40, **c**), and PEG- (PEG D_h_ 28, **d**) coated Mg doped γFe_2_O_3_ MNs measured at the different dispersion media. (**e**,**f**) The measured ΔT (**e**) and SLP (**f**). (**g**) The hydrodynamic size change of dextran-110 k, dextran-10 k, and PEG-coated Mg doped γFe_2_O_3_ MNs in dispersion media with various biological conditions.

The effects of physiological factors on the SLP and ΔT of MNs are critical to interpret and predict the heating performance in biological environments (e.g., tumor tissues) during MNFH. To precisely estimate the heating performance of MNs in biological environments, the AC heat induction behavior under the various physiological conditions were systematically explored. The salt concentration and pH are first considered as basic physiological factors since they can cause MNP aggregation. The aggregated MNPs increase the hydrodynamic size (*D*_*h*_) [effective volume (*V*_*h*_)] of MNs, hence the longer Brownian relaxation time constant, *τ*_*B*_, is induced as described in Eq. (4)^10,22^. This directly results in the reduction of SLP and ΔT.

4 $$\tau_{B} = \frac{{3\eta V_{h} }}{{K_{B} T}},$$

where, *η* is viscosity of dispersion medium, *K*_*B*_ is Boltzmann constant, and *T* is absolute ambient temperature. In addition, the aggregated MNPs affect the change of the Nèel relaxation time constant, *τ*_*N*_, by the aggregation-induced dipole interaction between the MNPs^12–15^. To comprehensively evaluate the heating power of MNs in the biological environments, the dextran-110 k coated Mg doped γFe_2_O_3_ MNs (*D*_*h*_ = 30 nm) were dispersed in water, 40% (wt.) glycerol solution, and 1% agarose gel with pH 5 and 150 mM NaCl to mimic the representative physiological environments of tumors (pH5, 150 mM NaCl, viscosity = 2.78 mPa‧s at 37 °C)^23,24^ (Fig. 4a). The dextran-10 k and polyethylene glycol (PEG)-coated Mg doped γFe_2_O_3_ MNs with *D*_*h*_ = 40 nm, and *D*_*h*_ = 28 nm, respectively, were also used for comparison. The dextran-110 k coated Mg doped γFe_2_O_3_ MNs (Dextran D_h_ 30) showed similar heat induction curves (Fig. 4b) and ΔT (Fig. 4e, grey, ~ 18 °C) independent of media, but the SLP was decreased at 40% glycerol solution (78 W g^−1^) and 1% agarose gel (65 W g^−1^) compared to water medium (100 W g^−1^) (Fig. 4f, grey). In contrast, the dextran-10 k coated Mg doped γFe_2_O_3_ MNs (Dextran D_h_ 40, Fig. 4c,e) showed a lower ΔT (~ 13 °C) and SLP (~ 65 W g^−1^), but had almost the same heat induction curves independent of the media. For PEG-coated Mg doped γFe_2_O_3_ MNs (PEG D_h_ 28), it showed relatively higher ΔT (22 °C) and SLP (98 W g^−1^) in the water compared to dextran coated Mg doped γFe_2_O_3_ MNs. However, the ΔT and SLP were very small and almost unmeasurable at 40% glycerol solution and 1% agarose gel conditions (Fig. 4d,e (green), and f (green)). As the heating power of MNs is dominantly determined by the *τ*_*B*_, the *D*_*h*_ of the three MNs in different dispersion medium with various pH and NaCl/glycerol was systematically investigated. The PEG-coated Mg doped γFe_2_O_3_ MNs showed the abrupt change of *D*_*h*_ in the range of 28–600 nm (Fig. 4g). Particularly, they showed severe aggregation in the salt-contained medium. However, the *D*_*h*_ of dextran-110 k (Fig. 4g, grey) and dextran-10 k (Fig. 4g, orange) coated Mg doped γFe_2_O_3_ MNs were stable at the same conditions. Accordingly, the aggregated MNs in higher salt-contained medium showed very low ∆T (Supplementary Fig. 7). These results indicate that the heating power of MNs is strongly influenced by the dispersion medium and surface coating status related to the colloidal stability. The PEG-coated Mg doped γFe_2_O_3_ MNs showed the maximum ΔT and SLP in the water due to the smaller *D*_*h*_ since the molecular weight of PEG (591–723 g mol^−1^) which is much smaller than that of dextran. However, they almost lost their heating capability at the salt-contained medium due to the severely increased *D*_*h*_. Because of the relatively large size of Mg doped γFe_2_O_3_ MNs (25 nm), the PEG molecules may not stably enclose the surface and easily aggregated at the salt-contained medium. In contrast, the dextran-110 k coated Mg doped γFe_2_O_3_ MNs showed superior heating power since their colloidal stability is outstanding in all the tested dispersion media. The ~ 25% decreased SLP in 40% glycerol solution is understood not by the colloidal stability but the viscosity difference between the water (0.7 mPa‧s at 37 °C) and the 40% glycerol solution (2.7 mPa‧s at 37 °C)^25^. For the dextran-10 k coated Mg doped γFe_2_O_3_ MNs, it may not stabilize the surface, hence aggregate up to 40 nm during the surface modification due to the relatively short length of dextran polymer. Therefore, the *D*_*h*_ was increased up to 30% compared to the dextran-110 k coated MNs directly resulting in the lower SLP. It was clearly demonstrated that the SLP and ΔT of MNs strongly depend on the testing sample container and physiological factors-induced change of colloidal stability in biological environments. Based on all the experimental results, the bio-mimicking model composed of, (1) sphere shape container, (2) 40% glycerol solution with pH 5 and 150 mM NaCl, and (3) water surrounding medium, is found to be the most likely condition to describe the tumor condition in-vivo. Therefore, we propose this condition as “Pseudo-Tumor Environment System (P-TES)” to reliably evaluate the ΔT and SLP behavior of MNs to directly predict the tumor temperature during MNFH.

### Verification of P-TES

**Figure 5:**
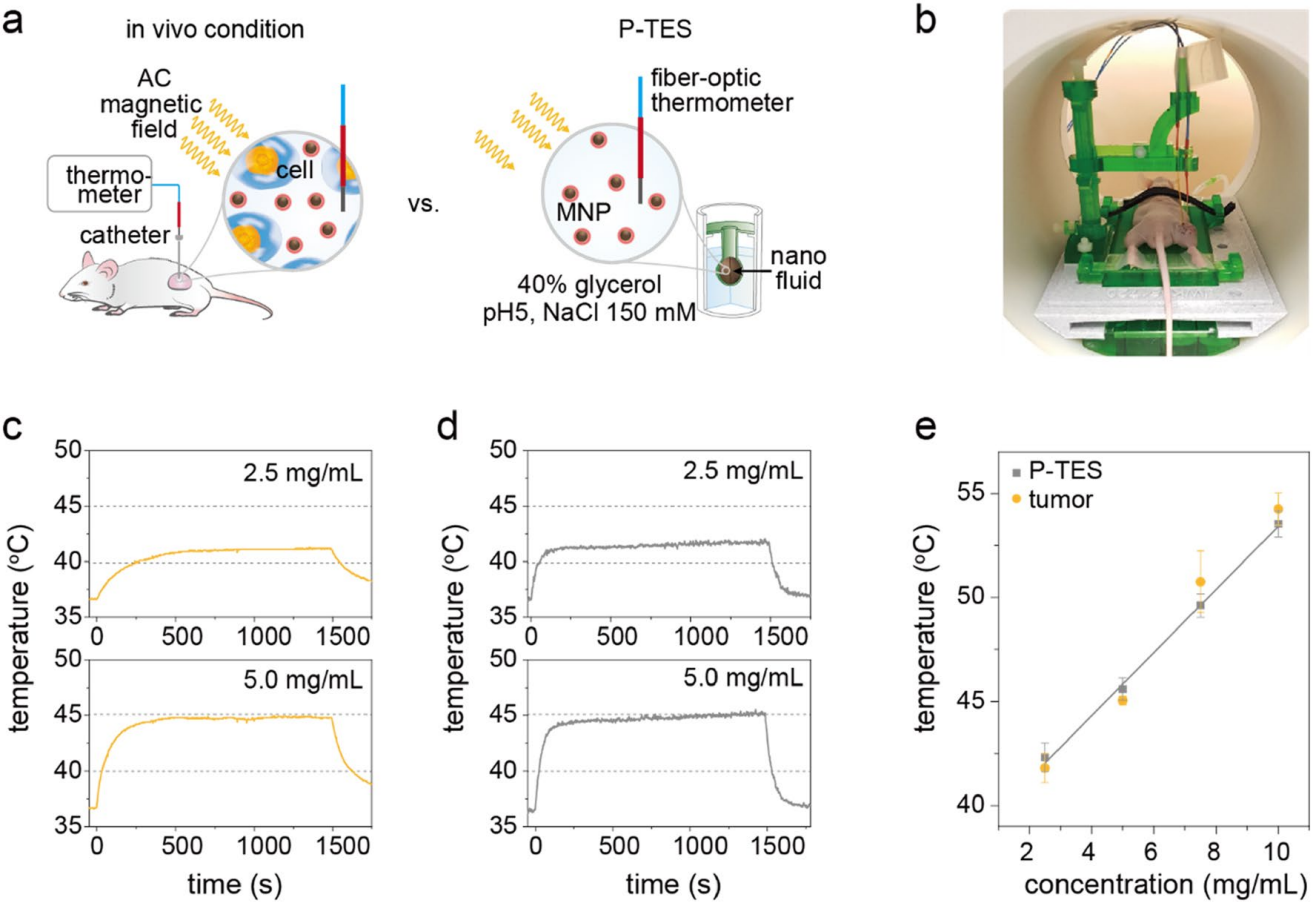
Verification of P-TES for MNFH application. (**a**) Schematic illustrations showing the comparison between the in vivo environment and the P-TES during MNFH. (**b**) A picture of experimental setup of in vivo real-time monitoring system for MNFH. (**c**,**d**) AC temperature rising curve measured at a tumor xenograft mouse model (**c**) and P-TES (**d**). (**e**) The comparison of maximum temperature during MNFH. In vivo animal model, orange; P-TES, grey.

To verify the reliability and accuracy of P-TES, the heat induction characteristics measured using P-TES were compared to a tumor xenografted mouse model (Fig. 5a). An estimated mean concentration of 2.5, 5.0, 7.5, and 10 mg mL^−1^ of dextran-110 k coated Mg doped γFe_2_O_3_ MN were injected to a tumor (volume, 0.3 mL). For comparison, the container volume of bio-mimicking phantom was fixed at 0.3 mL and the same amount of MNs were filled (see experiment conditions). The ${\text{H}}_{\text{AC,appl}}$ (*f*_*appl*_ = 100 kHz, *H*_*appl*_ = 140 Oe) was applied for 25 min and the real-time temperature changes were monitored at the central region of the tumor (Fig. 5b). Surprisingly, the T_AC,max_ measured at the tumor was accorded well to that from P-TES at the same concentration (Fig. 5c–e and Supplementary Fig. 8a,b). A slower dT/dt was observed at the xenografted tumor, but this is thought to be due to the inhomogeneous distribution of MNs in the tumor at the initial stage of MNFH (< 5 min). The comparison results shown in Fig. 5 clearly demonstrate that the proposed P-TES can accurately estimate the AC heating temperature of MNs, thus reliably predicting the heating temperature of MNs in tumors (tumor tissues) during MNFH.

In summary, P-TES to accurately estimate the heating capability of MNs is proposed and its biotechnical feasibility was demonstrated to reliably predict the ΔT in tumors during MNFH. Additionally, a simple and accurate method based on the Newton-Richman law to estimate the ΔT of MNs at in-vivo conditions using the SLP measured in the air was introduced to establish a ΔT prediction model. According to the analyzed results on the effects of external/internal experimental factors on the heating characteristics of MNs, a bio-mimicking phantom composed of: (1) sphere shape container, (2) 40% glycerol solution with pH 5 and 150 mM NaCl, and (3) water SM, was revealed as the P-TES condition to most accurately predict the ΔT of MNs in tumors during MNFH. Moreover, the ΔT of MNs determined by the ΔT prediction model was coincident well with the ΔT measured in tumors during MNFH. All the experimental results strongly demonstrate the proposed P-TES including ΔT prediction model can be recommended as a standardized measurement method of ΔT of MNs for direct clinical MNFH application. Moreover, the proposed P-TES and ΔT prediction model are expected to save time for screening the performance of newly developed MNFH agent and minimize the over-use of animals dramatically.

## Methods

### Materials and apparatus

Mg acetate tetrahydrate, Fe acetylacetonate, oleic acid, oleyl amine, benzyl were purchased from MiliporeSigma. Dextran (M_w_ = 10 kDa and 110 kDa) and trimethoxysilane PEG (M_w_: 591–723 g mol^−1^) were purchased from Phamacosmos A/S and Gelest for the surface modification. For the purification and concentration of MNPs, Amicon^®^ Stirred Cells (500 kDa, MilliporeSigma) was used. The Hypertheranoid™ 140 AC magnetic field generator (Neo-Nanomedics., Inc.) was used for measuring the AC heat induction characteristics. A 4-channel benchtop fiber-optic thermometer (FOTEMP1-4, Optocon^®^) and the FLIR One pro thermal camera (FLIR) were used for the temperature monitoring of magnetic nanofluid. All parts for motorized lifting stages (PT3 lifting stage, Z825B actuator, KST101 controller) were purchased from Thorlabs. All glassware was purchased from KJ LAB. Custom-built sample containers, water circulation system, and heat baths were fabricated using a 3D printer (Photon S, Anycubic; Digilab 3D45, Dremel).

### Synthesis of Mg doped γFe_2_O_3_ MNs

The Mg doped γFe_2_O_3_ MNs were synthesized based on the modified thermal decomposition method. For the synthesis of Mg doped γFe_2_O_3_ MNs, Mg acetate tetrahydrate (0.13 mmol), Fe acetylacetonate (2.0 mmol), oleic acid (1.2 mmol), and benzyl ether (20 mL) were added in a 50 mL round-bottom flask. The mixed reaction solutions were heated up to 200 °C for 30 min (~ 8 °C min^−1^) and maintained for another 50 min (nucleation step) under N_2_ gas at the flow rate of ~ 100 mL min^−1^. Then, the solutions were heated again up to 296 °C for 20 min (~ 5 °C min^−1^) and maintained for 60 min. After cooling down to room temperature, the product was washed with 100 mL of toluene 3 times. A NdFeB magnet was used for washing and purification of Mg doped γFe_2_O_3_ MNs. The final product was dispersed in 100 mL of toluene.

### AC magnetically-induced heating of MNs

The AC heat induction of MNs was characterized using an AC magnetic field induction system. For measuring the temperature distribution of nanofluid using a fiber-optic thermometer, the AC magnetic field generator with a 50 mm (diameter) vertical AC magnetic coil (Supplementary Fig. 1) was used. The AC magnetic field generator with a 140 mm (diameter) horizontal AC magnetic coil (Supplementary Fig. 4 and 5) was used for thermal imaging of the sample container during AC heat induction. The sample container (volume of nanofluids: 1 mL) was placed in the center of AC coil to measure the AC magnetically induced heating properties. The AC heat induction temperature was monitored using a fiber-optic thermometer. The sampling rate was 1 point per second. The *H*_*appl*_ and *f*_*appl*_ were fixed at 140 Oe and 100 kHz, respectively. The SLP value of all the Mg doped γFe_2_O_3_ MNs were calculated based on the following equation of $\text{SLP }\text{(W/g)} \, = \text{ } \frac{{\text{C}}{\text{V}}_{\text{s}}}{\text{m}}\frac{\text{dT}}{{\text{dt}}}$, which is described in the introduction section.

### Preparations of glycerol and agarose phantom

The MNs were dispersed in glycerol and agarose gel to test the heat induction power in pseudo biological environments. (1) glycerol phantom: A 75 μL of 2 M NaCl and a 25 μL of 1 M acetate buffer solution (pH5) were added to 0.5 mL of 20 mg mL^−1^ Mg doped γFe_2_O_3_ MN. Then, a 400 mg of glycerol was added to the mixture solution and shake for 1 min. The final concentration of glycerol and NaCl were 40% (wt) and 150 mM, respectively. (2) Agarose phantom: A 400 mg of agarose, a 250 mg of NaCl, and a 1 mL of 1 M acetate buffer solution (pH5) were added to 19 mL of deionized water. A mixture was heated up using a microwave until the agarose is completely dissolved. Rapidly transfer a 0.5 mL of mixture solution to a 0.5 mL of 20 mg mL^−1^ Mg doped γFe_2_O_3_ MN that contained a sample tube. Cooldown to the mixture for 1 h. The final concentration of agarose, and NaCl were 1%, and 150 mM, respectively.

### In vivo MNFH experiment

All animal experiments were conducted with the guideline of the Association for Assessment and Accreditation of Laboratory Animal Care International (Frederick, MD, USA) and under the approval of the Institutional Animal Care and Use Committee (Augusta University, GA, USA, Reference number: 2016-0803). All methods used for in vivo studies in mice are in accordance with ARRIVE guidelines. The LNCaP xenograft mice were prepared for in vivo MNFH studies. Briefly, 2 × 10^6^ cells of LNCaP were implanted in the right thigh of athymic nude mice (Balb/c nude). When the tumor volume grew more than 0.25 mL, the mice were divided into 4 groups and 0, 2.5, 5.0. 7.5, and a 10 mg/mL_tumor_ of Mg doped γFe_2_O_3_ MNs were injected. The nanofluids were injected into the tumor directly using a syringe pump with an injection speed of 5 µL min^−1^. The volume of the tumor was 0.25–0.35 mL and the injected volume of MN was around 70 µL. Then, mice were placed on the 37 °C heating bed to keep the body temperature and the fiber-optic thermometer was mounted to the center of the tumor for real-time tumor temperature measurement. MNFH (*f*_*appl*_: 100 kHz, *H*_*appl*_: 140 Oe) was carried out for 25 min and the tumor temperature was recorded.

## Supplementary Information

Supplementary Information.
