# Supplementary material for: Reliable evaluation method of heating power of magnetic nanofluids to directly predict the tumor temperature during hyperthermia
Source: Sci Rep. 2021 Nov 11;11:22028. doi: 10.1038/s41598-021-01321-3 (PMC8586151; doi:10.1038/s41598-021-01321-3)
Supplement: Supplementary file 1 — Supplementary Information. [file 41598_2021_1321_MOESM1_ESM.docx]

*Supplementary information*

***Reliable evaluation method of heating power of magnetic nanofluids to directly predict the tumor temperature during hyperthermia***

Ji-wook Kim^1^ and Seongtae Bae^1,2^*

^1^Nanobiomagentics and bioelectronics laboratory (NB^2^L), Department of Electrical Engineering, University of South Carolina, 301 Main Street, Columbia, SC 29208, USA

^2^Biomedical engineering program, College of Engineering and Computing, University of South Carolina, 301 Main Street, Columbia, SC 29208, USA

*Corresponding author

E-mail: [bae4@cec.sc.edu](mailto:bae4@cec.sc.edu)

**Supplementary methods**

**X-ray diffraction patterns of Mg doped γ-Fe_2_O_3_ MNPs** The crystal structure was analyzed using a Cu-Kα radiated X-ray diffractometer. All the synthesized Mg doped γFe_2_O_3_ MNPs showed a single-phase cubic spinel ferrite structure and did not exhibit any undesirable crystalline phases. All the X-ray diffraction patterns of Mg doped γFe_2_O_3_ MNPs were well indexed and correlated to those of typical cubic spinel structures (JCPDS #38-0430)

**Surface modification of Mg doped γFe_2_O_3_ MNPs** For dextran coating, a 5 mL of as-synthesized Mg doped γFe_2_O_3_ MNPs were treated with 4 mg of tetramethylammonium hydroxide (TMAOH) for 5 hours at the 70^o^C water bath. The MNPs were magnetically separated using NdFeB magnet and washed with a 5 mL of acetone for two times. A 15 mL of 100 mg/mL dextran solution was added and the mixture was heated up for 12 hours. After the reaction, the dextran-coated Mg doped γFe_2_O_3_ MNPs were purified by Amicon® Stirred Cells. For PEG coating, a 1 mL of as-synthesized Mg doped γFe_2_O_3_ MNPs was mixed with a 4 mL of 0.8 M TMAOH/methanol solution and a 0.3 mL of trimethoxysiliane PEG. The mixture was sonicated for 8 hours at 60^o^C. After the reaction, the PEG-coated Mg doped γFe_2_O_3_ MNPs were purified by magnetic separation (NdFeB magnetic) and the final product was dispersed in deionized water.

**DC magnetization measurement** The DC M-H loops (major and minor loops) were measured using a vibrating sample magnetometer (VSM). The MNs (10 mg/mL, 0.08 mL) was loaded to the sample holder. Demagnetization field was applied before measurement. DC major and minor hysteresis loop of Mg doped γFe_2_O_3_ MNs nanofluid measured at the sweeping field of ± 5 kOe, and ± 150 Oe, respectively.

**Supplementary Figures**

**
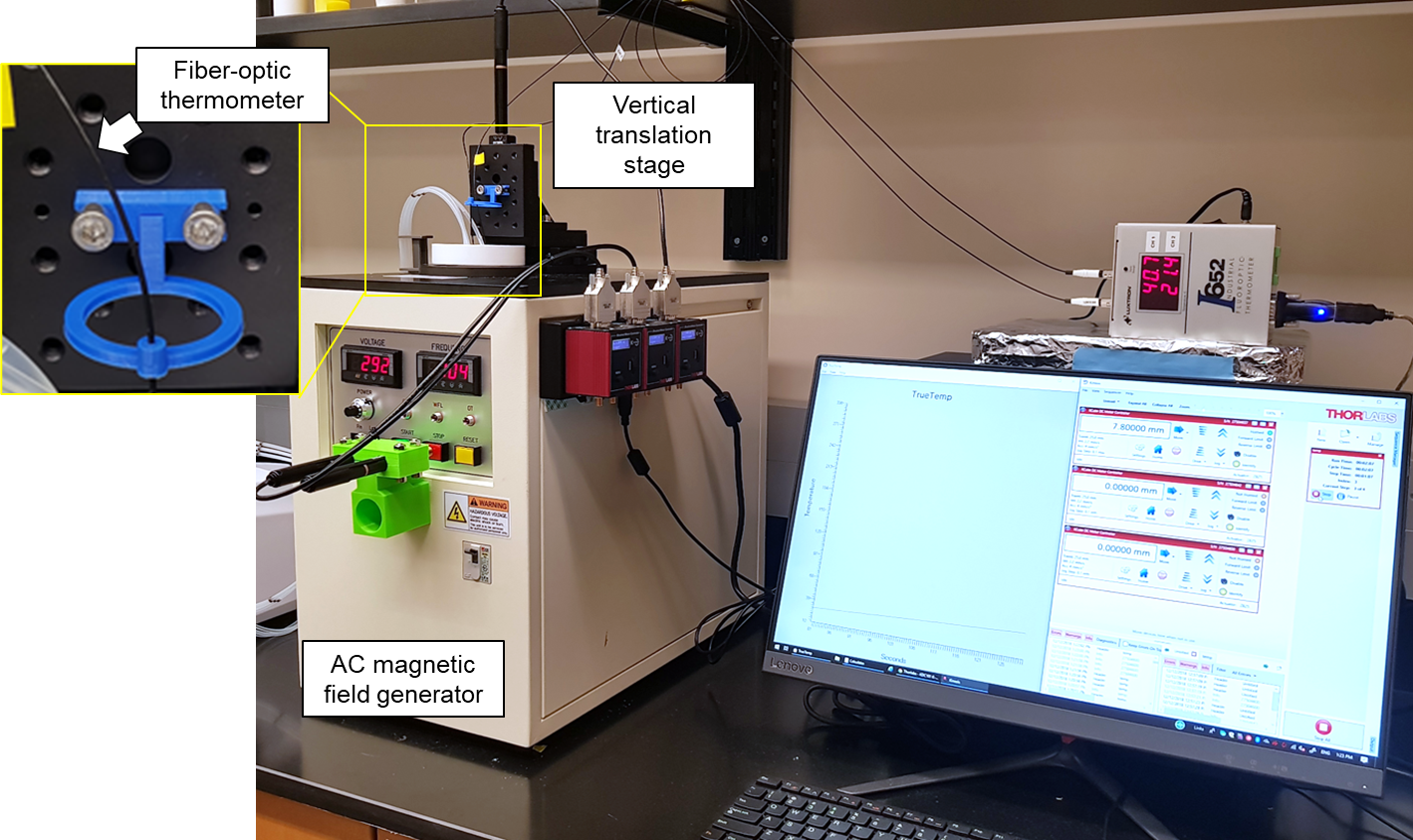
**

**Supplementary Figure 1.** The vertical translation stage and fiber-optic thermometer mounted at 50 mm (diameter of AC magnetic coil) AC magnetic field generator.

**
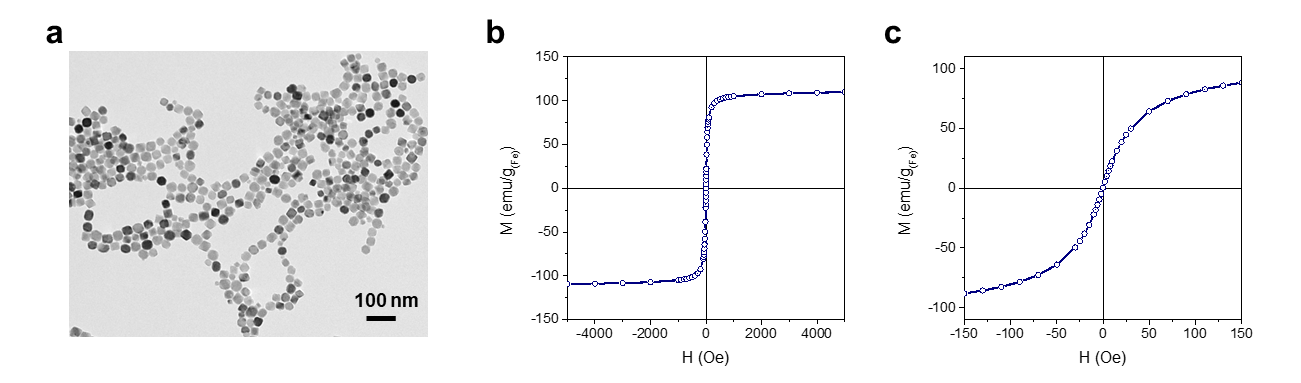
**

**Supplementary Figure 2.** **a**, A TEM image of 25 nm Mg_x_-γFe_2_O_3_ MNPs with σ < 10% (hydordynamic diameter, *D_h_* = 35 nm in magnetic nanofluids). **b,c**, Major (b) and minor (c) M-H loop of Mg doped γFe_2_O_3_ nanofluid measured at the sweeping magnetic field of ±5000 Oe and ±150 Oe, respectively. 20 mg_Fe_/mL (80 μL) of Mg doped γFe_2_O_3_ MNs was used for the VSM measurement.


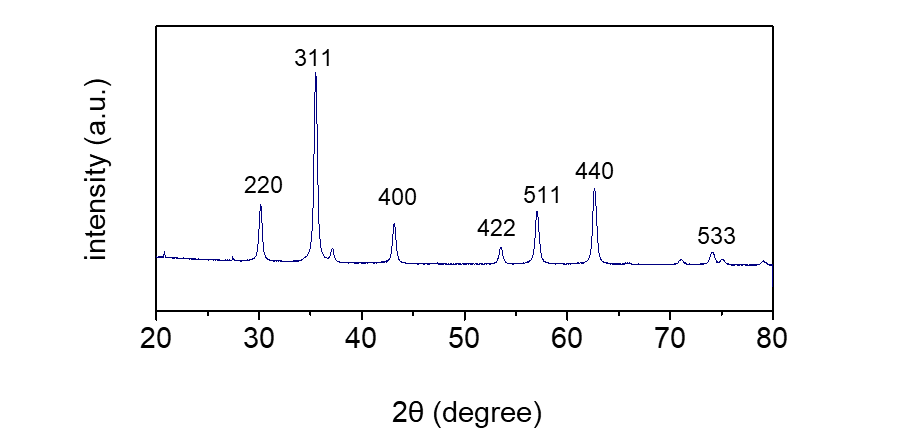


**Supplementary Figure 3.** The X-ray powder diffraction analysis of Mg doped γFe_2_O_3_ MNP.

**
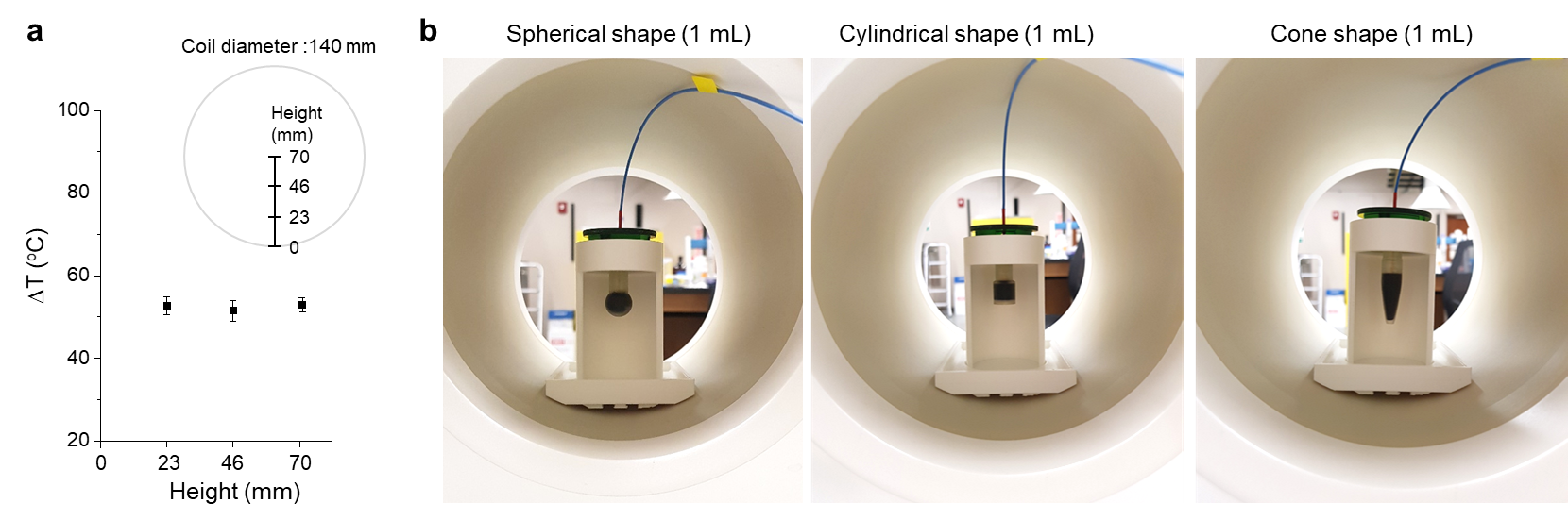
**

**Supplementary Figure 4.** **a**, AC magnetic heat induction (∆T at 300 s) of Mg doped γFe_2_O_3_ MN measured at the different heights in the AC magnetic coil. Sample containers, spherical shape; concentration of MN: 10 mg/mL. A negligible ∆T change was observed at the height range of 23 ~ 70 mm where the spherical, cylindrical, and cone shape containers are placed. This result represents that there is a negligibly small deviation of AC magnetic field strength along the both vertical and longitudinal directions. **b,** Pictures of experimental setups for obtaining thermal imaging of the sample container (spherical, cylindrical, and cone shape) during the application of AC magnetic field.

**
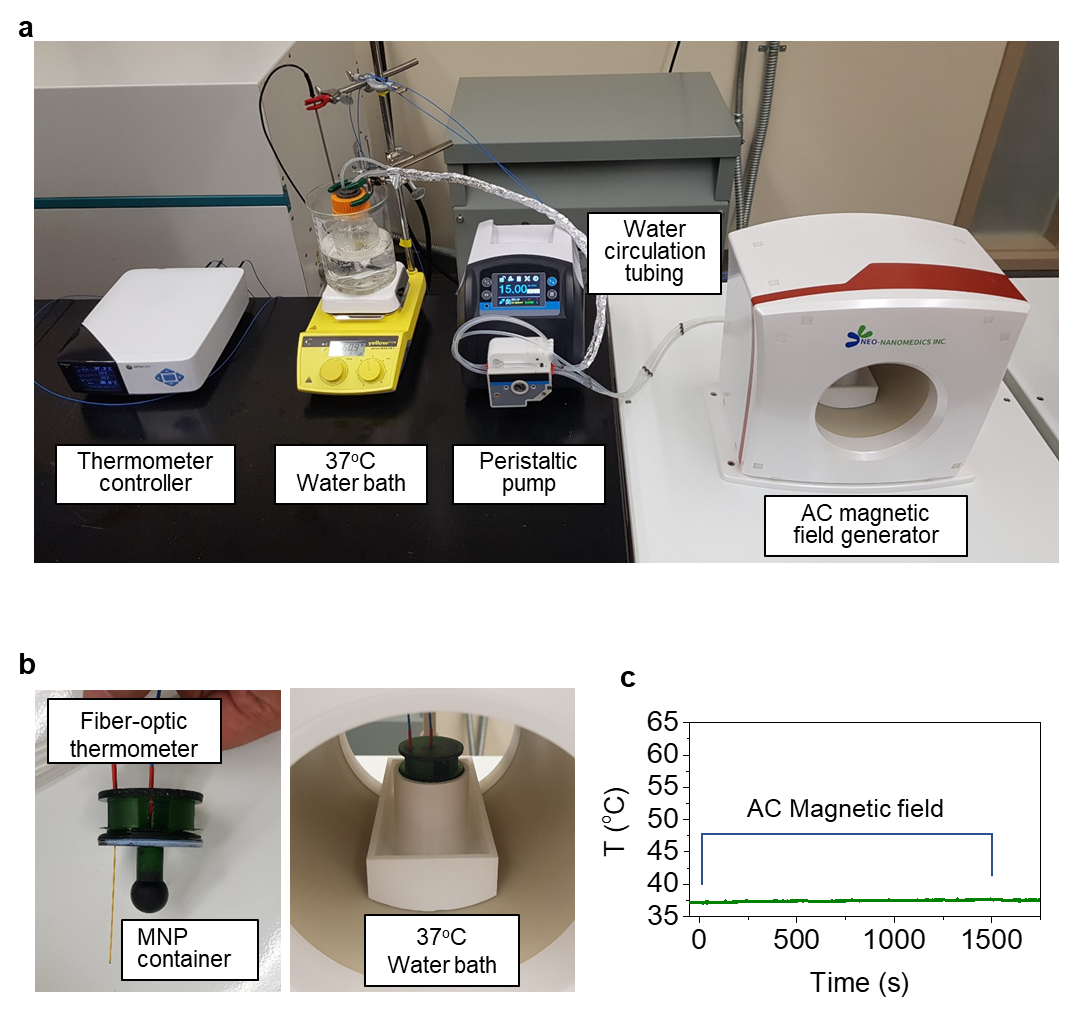
**

**Supplementary Figure 5.** **a**, A 140 mm (diameter of AC magnetic coil) AC magnetic field generator with a thermometer, temperature controlling system (water bath), and circulation system (peristaltic pump). **b**, The sample container with fiber-optic thermometers (left) and water bath for containing surrounding medium (right). Two fiber-optic thermometers were placed in the sample container for measuring inside (nanofluid) and outside (surrounding medium) temperature of the container. **c**, The temperature of surrounding medium during the application of AC magnetic field.

**
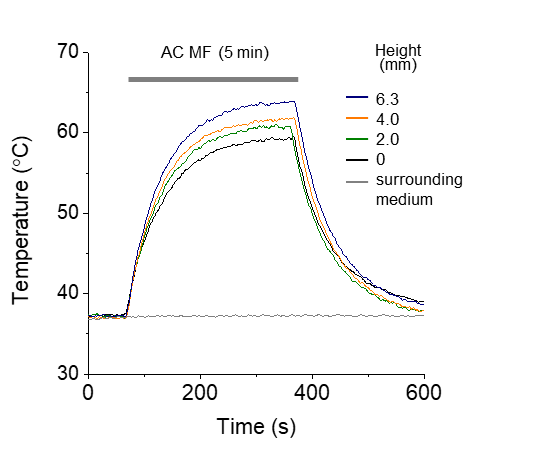
**

**Supplementary Figure 6.** The heat induction curve of Mg doped γFe_2_O_3_ MN measured at the different positions (heights) of sphere-shape sample container (diameter, 6.3 mm). The height indicates a vertical distance from the bottom (0 mm) to the fiber-optic thermometer probe in the sample container.


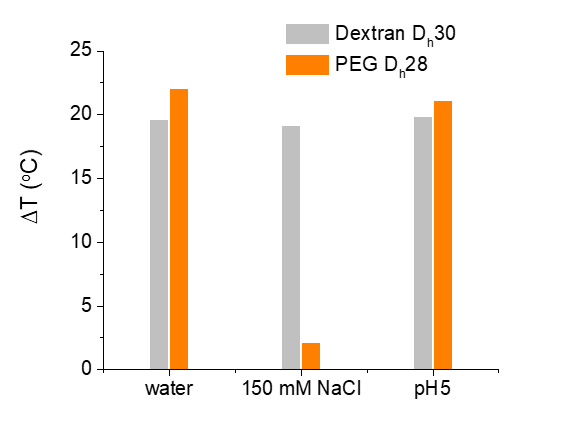


**Supplementary Figure 7.** Heat induction (∆T at 300 s) of dextran-110 k (Dextran D_h_ 30) and PEG (PEG D_h_ 28) coated Mg doped γFe_2_O_3_ MN measured in the water, 150 mM NaCl solution, pH 5 citrate buffer solution. Surrounding medium, 37^o^C water.


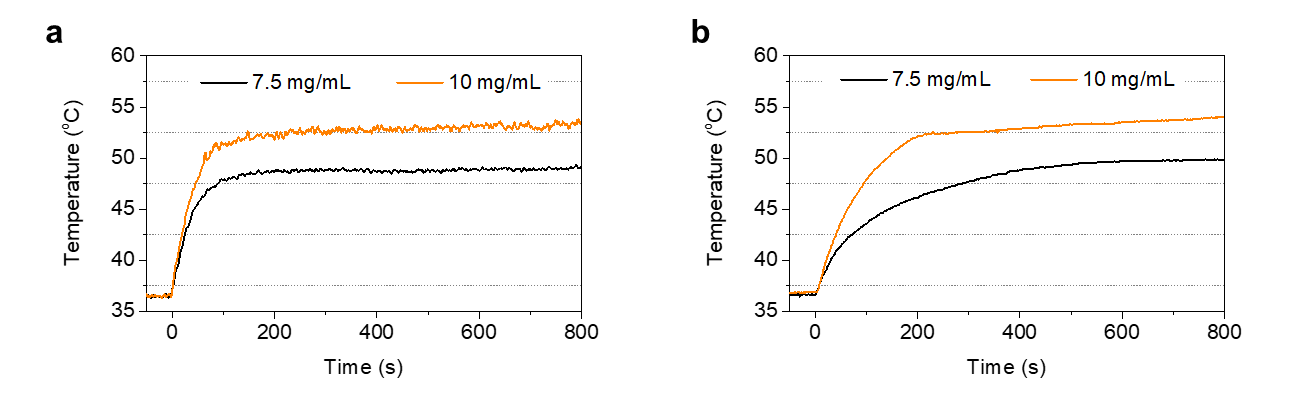


**Supplementary Figure 8.** The heat induction curves of Mg doped γFe_2_O_3_ MNs measured at P-TES (a) and tumor-xenografted mouse models (b).
